# Asiaticoside Increases Caspase-9 Activity in MCF-7 Cells and Inhibits TNF-α and IL-6 Expression in Nude Mouse Xenografts via the NF-κB Pathway

**DOI:** 10.3390/molecules28052101

**Published:** 2023-02-23

**Authors:** Fatma J. Al-Saeedi

**Affiliations:** Department of Nuclear Medicine, College of Medicine, Kuwait University, P.O. Box 24923, Safat 13110, Kuwait; fatma.alsaeedi@ku.edu.kw; Tel.: +965-24636409

**Keywords:** asiaticoside, MCF-7 cells, nude mice, tumor, apoptosis, cytotoxicity, NF-κB

## Abstract

**Background:** We hypothesized that the antitumor effects of asiaticoside on breast cancer are driven by its ability to decrease the expression of tumor inflammation-promoting genes and increase apoptotic signaling. In this study, we aimed to better understand the mechanisms of action of asiaticoside as a chemical modulator or as a chemopreventive agent in breast cancer. **Methods**: MCF-7 cells were cultured and treated with 0, 20, 40, and 80 μM asiaticoside for 48 h. Fluorometric caspase-9, apoptosis, and gene expression analyses were conducted. For the xenograft experiments, we divided nude mice into the following 5 groups (10 animals per group): group I, control mice; group II, untreated tumor-bearing nude mice; group III, tumor-bearing nude mice treated with asiaticoside at weeks 1–2 and 4–7 and injected with MCF-7 cells at week 3; group IV, tumor-bearing nude mice injected with MCF-7 cells at week 3 and treated with asiaticoside beginning at week 6; and group V, nude mice treated with asiaticoside, as a drug control. After treatment, weight measurements were performed weekly. Tumor growth was determined and analyzed using histology and DNA and RNA isolation. **Results:** In MCF-7 cells, we found that asiaticoside increased caspase-9 activity. In the xenograft experiment, we found that TNF-α and IL-6 expression decreased (*p* < 0.001) via the NF-κB pathway. **Conclusion:** Overall, our data suggest that asiaticoside produces promising effects on tumor growth, progression, and tumor-associated inflammation in MCF-7 cells as well as a nude mouse MCF-7 tumor xenograft model.

## 1. Background

In 2020, there were 2.3 million women identified with breast cancer and more than 650,000 associated deaths worldwide. This makes it the world’s most prevalent cancer [1,2,3].

There are many strategies to treat breast cancer such as surgery, chemotherapy, hormonal, biological therapy, immunotherapy, and radiation therapy. The limitations include recurrence resistance and variations in patients’ response. The main obstacle for all of these treatments is the systemic toxicity. Chemoprevention overcomes the systemic damage or toxicity and can provide strong inhibition or delay the initiation of malignancy or cancer. Chemoprevention is defined as the use of specific chemicals or natural compounds or substances to aid lower a person’s possibility of developing cancer or prevent it from resuming or treating it.

There are many chemopreventive agents that utilize different mechanisms to block, suppress, delay, and treat cancer [4,5] such as Nrf2 signaling pathways [6], signal transducers [7], and activators of transcription 3 [8].

One of the rather promising agents of chemoprevention is the substance called asiatiscoside. Asiaticoside is one of the major active compounds acquired from a plant called *Centella asiatica* and was extensively reported and proven to share a lot of pharmacological and medicinal tasks. Many studies reported that asiaticoside expressed anti-inflammatory [9] and different effects in multiple human disease models [10]. Moreover, asiaticoside has shown anti-cancer effects in a series of human malignancies, including multiple myeloma [11], melanoma [12], glioma [13], hepatic [14], and breast cancer [9,15].

In our laboratory, the effects of asiaticoside on MCF-7 cells and on 7,12-dimethylbenz(a)anthracene (DMBA)-induced rat cancer models were investigated. The half-maximal inhibitory concentration (IC50) was reported at 40 μM asiaticoside in a dose dependent manner. In addition, we reported that caspase-3 activity increased, and the expression of the cytokines TNF-α and IL-1β was significantly decreased in MCF-7 cells treated for 48 h asiaticoside. The uptake pattern of asiaticoside in MCF-7 cells after 48 h treatment was documented using lipophilic tumor imaging agents. ^99m^Tc-tetrofosmin (^99m^Tc-Tfos) and ^99m^Tc-sestamibi (^99m^Tc-MIBI) showed that asiaticoside inhibited 16% and 47% of ^99m^Tc-Tfos and ^99m^Tc-MIBI, respectively, in comparison to the control [16]. These studies showed that asiaticoside enhances the antitumor activity in MCF-7 cells and acts as a biochemical modulator.

The nuclear factor kappa-light-chain-enhancer of activated B cells (NF-κB) is a protein complex that controls many cellular processes such as inflammation, cytokine production, cell proliferation, survival, and immune response to infection and cancer [17,18]. The signaling pathways of the NF-κB are manifested through the association between NF-κB dimers, IκB regulators, and IKK complexes. Different stimuli and suppressors can be shown according to a predefined signal [19]. Several studies reported that NF-κB suppresses aging, inflammation, and cancer.

To this end, we aimed to examine pathways of inflammation stimuli to NF-κB, such as tumor necrosis factor-α (TNF-α) and interleukin 1 beta (IL-1β). In addition, studying the antitumor effects of asiaticoside, discovering the mechanism of action of asiaticoside regulation of caspase-9 in MCF-7 cells, and exploring an MCF-7 xenograft nude mouse model were also aimed for. There is little research on asiaticoside and caspase-9 action. In this study, asiaticoside and caspase-9 will be investigated.

## 2. Results

### 2.1. Cell Viability (MTT) Assay

**Figure 1:**
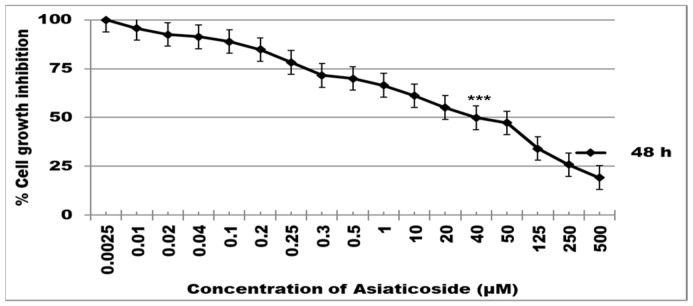
The effect of asiaticoside on cell viability. MCF-7 cells were incubated at 37 °C with several concentrations of asiaticoside for 48 h. The cytotoxicity of asiaticoside was measured using an MTT assay. *** *p* < 0.0001 one-way ANOVA. Values are mean ± SD from six independent experiments.

Figure 1 shows the percentage inhibition of MCF-7 cell growth for a 48 h asiaticoside incubation period. The half maximal inhibitory concentration (IC50) was determined to be 40 μM of asiaticoside using the MTT assay. Asiaticoside is toxic at this concentration, as demonstrated by the significant reduction in cell viability (one-way ANOVA: F = 22.31, *p* < 0.0001; n = 6 for each different concentration).

### 2.2. Asiaticoside Treatment Increases Caspase-9 Activity in MCF-7 Cells

In this study, the IC50 40 µM of asiaticoside was used. To assess the effects of asiaticoside treatment on apoptosis, and to rule out the inhibition of caspase-9, here, caspase-9 protein expression was measured in the xenograft tumors. Caspase-9 protein expression was shown in groups II, III, and IV using the Western blot assay. Asiaticoside treatment of MCF-7 xenograft groups or cells significantly increased the protein expression of group III (*p* < 0.001) and group IV (*p* < 0.001) rather than group II.

**Figure 2:**
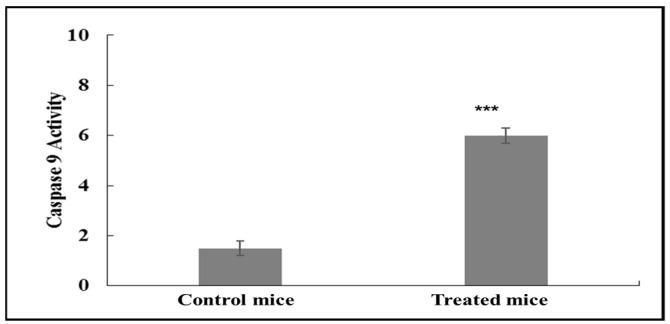
Activation of caspase-9 was measured by a fluorometric assay. Nude mice tumors treated with asiaticoside. Data represent the mean ± SD of six independent experiments. *** *p* < 0.0001 as compared to controls.

Figure 2 shows increased expression of caspase-9 activity in control samples and asiaticoside-treated mice. The stimulation of apoptosis by asiaticosde was demonstrated by an increase in caspase-9 (*p* < 0.0001) activity in samples treated with asiaticoside compared to the control group. Values were expressed as the mean ± SD from six independent experiments.

**Figure 3:**
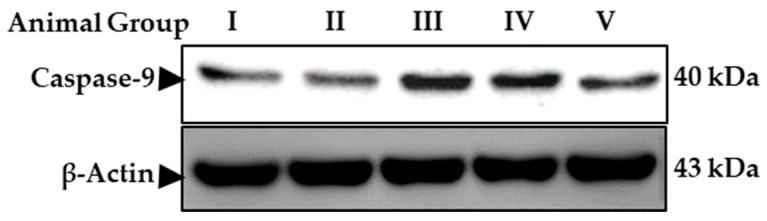
Western blotting of breast nude mice tumors collected in April 2018 using antibodies against caspase-9. Groups I–V.

Western blot analysis of samples 48 h after asiaticoside treatment showed a significant (*** *p* < 0.0001) increase in caspase-9 levels when the nude mice xenografts were pre-treated with asiaticoside (group III) and post-treated (IV group), compared to the control and II group. Values are mean ± SD from three independent experiments (Figure 3). Asiaticoside-induced apoptosis was measured by Western blotting of breast nude mice tumors collected using antibodies against caspase-9.

### 2.3. Detection of Apoptosis Using ^99m^Tc-Labeled Annexin V and MIBI 

**Figure 4:**
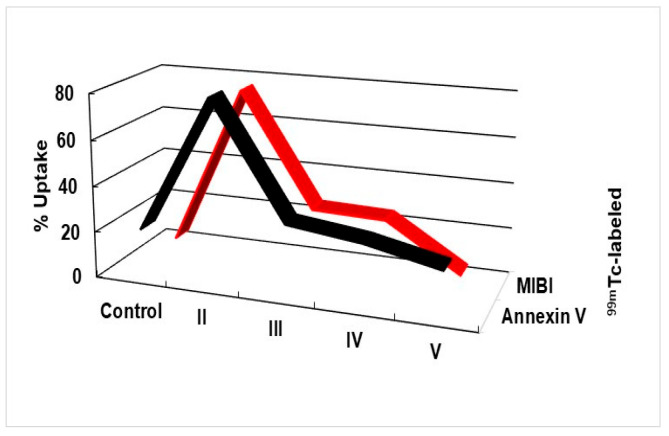
Correlation of 99 mTc-HYNIC-annexin V or 99 mTc-MIBI with MCF-7 cells underwent asiaticoside treatment for 48 h. Percentages of uptake are plotted with a best-fit line by linear regression analysis and are shown as (r^2^ = 0.97).

Figure 4 shows the effect of asiaticoside on different groups. Treated MCF-7 cells showed a strong correlation (*p* < 0.001) with ^99m^Tc-HYNIC-annexin V or ^99m^Tc-MIBI. The percentages of uptake are plotted with a best-fit line by linear regression analysis and are shown as (r^2^ = 0.97). Here, the in vitro radioactivity of radiolabeled-annexin V was compared with caspase-9. Gamma-counting results were expressed as the percentage of radioactivity bound to the apoptotic cells and were calculated as the following: % Bound = (A/A + B) × 100. A is the radioactivity recorded in the cell pellet, while B is the radioactivity in the supernatant.

### 2.4. Cytokines Detection Using ELISA

**Figure 5:**
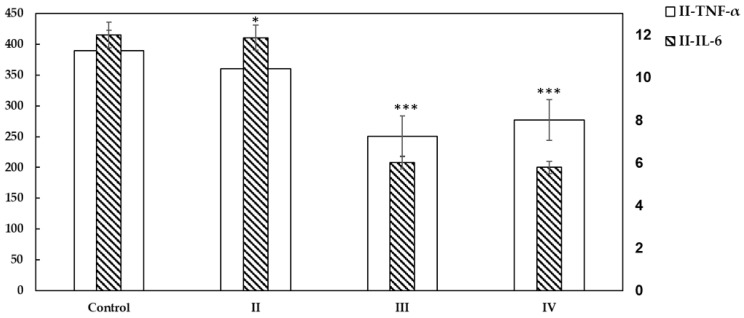
Protein expression of IL-6 and TNF-α in MCF-7 tumor xenografts. All experiments were repeated six times and data are presented as mean ± SD with error bars (n = 6), * *p* < 0.05; *** *p* < 0.001. An unpaired Student t-test was conducted in comparison to the control.

Figure 5 shows the protein expression levels of IL-6 and TNF-α expressed as optical density (O.D.) and reported as mean + SD in the studied groups II, III, and IV in comparison to the control. The results presented a highly significant decrease in IL-6 and TNF-α secretion in III and IV when they were treated with asiaticoside (*** *p* < 0.001) compared to the control and untreated group II. All experiments were repeated six times and data are presented as mean ± SD with error bars (n = 6, * *p* < 0.05; ** *p* < 0.01; *** *p* < 0.001 unpaired Student *t*-test).

### 2.5. Analyses of mRNA Expression

**Figure 6:**
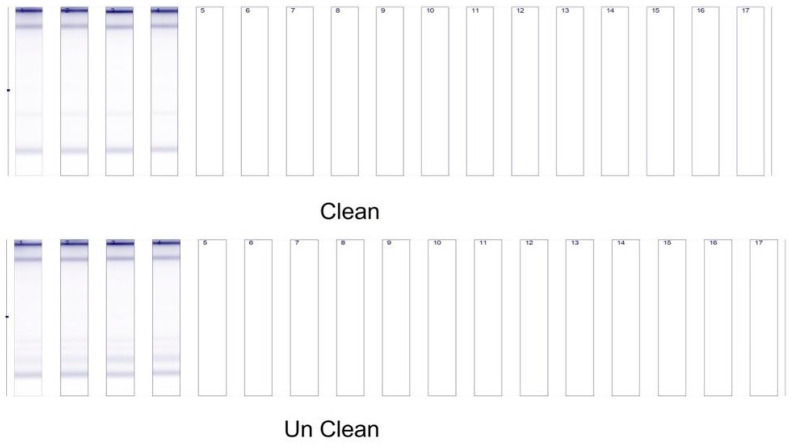
The tape station for cleaned and uncleaned extracted RNA samples using the RNAeasy micro kit for MCF-7 cells after 0, 20, 40, and 80 µM.

Figure 6 shows all extracted RNA samples that had good yields and good quality, as indicated by the Tape Station profiles for both raw and purified RNA. The Screen Tape Degradation Value (SDV) is a measure of RNA quality [20].

### 2.6. Tumor Formation and Growth in Xenograft Mice

**Figure 7:**
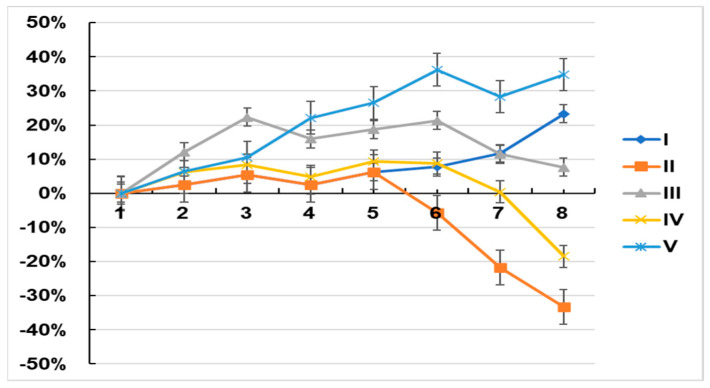
Data represent the mean ± SD of six independent experiments. ** *p* < 0.001 as compared to controls. The number of mice with tumors is 10 with four tumors per mouse. Mean values are body weights ± SD.

Group I and V (controls) mice gained weight throughout the experimental period, whereas those in groups II and IV (induced tumor and asiaticoside-treated induced tumor on week six groups, respectively) increased weight but began losing weight at week five and continued to lose weight up until the time of euthanasia. The weights of the mice in group III increased until week three, after which they remained stable until week six and then decreased until the experimental endpoint (eight weeks, Figure 7).

Here, the effect of asiaticoside as a chemical modulator or as a chemopreventive agent in breast cancer was assessed using an in vivo xenograft model as described above. Group III asiaticoside-pretreated induced tumors showed highly significant modulation and chemoprevention in body weight as compared to the control and among groups.

**Figure 8:**
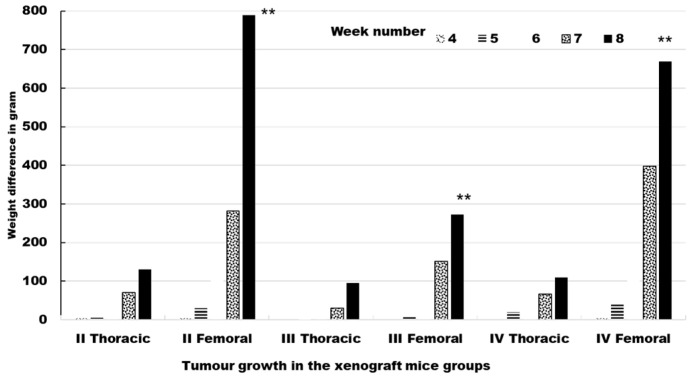
Tumor growth in the xenograft mice groups. Right injected thoracic and femoral sites. The number of mice with tumors is 10 with 4 tumors per mouse. Data represent the mean ± SD. ** *p* < 0.001 as compared to controls.

Four sites were injected in each mouse. The injected cells developed into palpable masses. Tumor formation started in week seven. The data relating to tumor growth in the xenograft mice are summarized in Table 1 (Figure 8). In groups II, III, and IV, all of the injected tumors grew, which allowed us to assess 120 tumors in total.

After the mice were euthanized at the end of week eight, only primary tumors were detected. Mice were examined by gross necropsy. In addition, samples were also examined by microscopy to evaluate the presence of tumor cells. Mice were evaluated for metastases. The regional and/or distant metastases were not found in animals.

#### 2.6.1. TNF-α and IL-6 Expression Is Reduced and Tumour Necrosis Is Increased in Xenograft Tumors from Mice Treated with Asiaticoside

The analysis of gene expression of the antitumor effect was performed to explore the mechanism related to asiaticoside. In MCF-7-tumour-bearing nude mice, an asiaticoside treatment dose of 150 mg/kg was administered intraperitoneally. The tumor tissue was evaluated after asiaticoside treatment. The expression of the cytokines IL-6 and TNF-α was highly significant decreased (*p* < 0.001) in group III and IV (Table 2).

#### 2.6.2. Activation of NF-kB Assay

**Figure 9:**
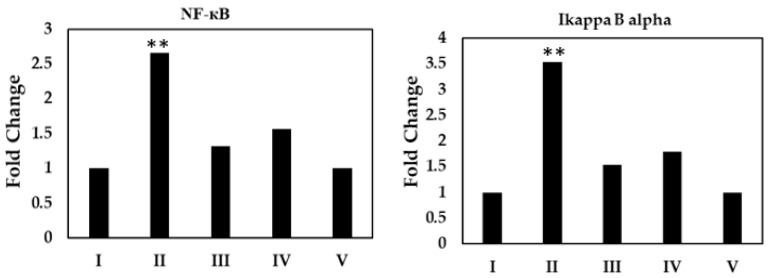
Immunoblot analysis of the inhibition of IκBα and NF-KB in xenografted whole mice tumors. ** *p* < 0.001 as compared to controls.

Asiaticoside inhibited the degradation of cytosolic inhibitory protein (IκBα), phospho-I-κ kinase (IKK), and NF-kB two-fold in comparison to the control levels (*p* < 0.001) using immunoblot analysis. This inhibition of degradation was differentiated from increased protein expression (Figure 9).

## 3. Methods and Materials

All chemicals and reagents used in this study were purchased from Sigma-Aldrich (UK). All immunohistochemistry primary and secondary antibodies were purchased from Cell Signaling Technology (USA). Asiaticoside (CAS:16830-15-2) was purchased from Sigma-Aldrich (UK). Antibodies targeting β-actin and nuclear factor κB (NF-κB) for Western blotting were purchased from Gibco (Thermo Fisher Scientific Inc., USA). A Nuclear Extract Kit (catalogue number 40010 and 40410) and Trans-AM NF-κB enzyme-linked immunosorbent assay (ELISA) kit were purchased from Active Motif (USA).

### 3.1. Preparation of Asiaticoside

Asiaticoside was prepared as a 1 mM stock in dimethylsulfoxide (0.1% DMSO) and serially diluted with Dulbecco’s modified Eagle medium (DMEM). 

### 3.2. Cell Culture

The Michigan Cancer Foundation-7 (MCF-7) cells, a breast cancer cell line, was purchased from Cell Lines Service (Germany) and cultured in DMEM supplemented with 10% fetal calf serum, 200 mg/mL streptomycin-penicillin, and 150 mg/mL L-glutamine. 

### 3.3. In Vitro Cell Experiments

The cells were seeded into 96-well plates at 2 × 10^5^ cells/mL. The cells were left to become confluent and then the plates were divided into two groups for treatment. The first group concerned using cells alone (control) while the other group concerned treating cells (asiaticoside). Asiaticoside was used for treatment at different concentrations (0.0025 to 500 µmol) and IC50 40 µmol was used in this study.

After the treatment of cells, the following was performed.

### 3.4. Fluorometric Assay Assessing Caspase-9 Activation and Apoptosis

MCF-7 cells were seeded in triplicate in 96-well plates (2.5 × 10^5^ per well), cultured overnight, and then treated with 40 µM asiaticoside for 48 h. Mitomycin C (40 µM), a known caspase-9 activator, was used as a positive control for caspase-9 activation. Caspase-9 assay solution was added to the cells. The activity of caspase-9 was assayed using the fluorogenic substrate calbiochem. Cell protein (50 mg) was extracted and incubated for 1 h with 400 nM reaction mixture (500 mL) containing 10 mM Hepes-NaOH, pH 7.4, 40 mM b-glycerophosphate, 50 mM NaCl, 2 mM MgCl_2_, 5 mM EGTA, 1 mM dithiothreitol, 2 mM ATP, 10 mM creatine phosphate, and 50 mg/mL of creatine kinase. The fluorescence intensity of extracts was read and measured on a plate reader over a range of 480 to 540 nm.

### 3.5. Cytokines Detection Using Enzyme-Linked Immunosorbent Assay (ELISA)

Cytokines were determined at the protein level using the ELISA method. MCF-7 cells were incubated with 40 µM asiaticoside for 48 h. To assess IL-6 and TNF-α, MCF-7 cells were added to the lysis buffer containing 27 mM Tris-HCl at a pH of 7.4, 145 mM NaCl, 1% NP-40, 1 mM EDTA, and 5% glycerol. Then, the cells were kept in ice for 10 min and centrifuged at 9000 rpm at 4 °C for 4 min. The upper layer was used to measure the level of IL-6 and TNF-α. Protein expression was detected using an ELISA kit following the manufacturer’s directions. 

### 3.6. Real-Time (RT)-PCR Analysis of Gene Expression in MCF-7 Cells

MCF-7 cells (5 × 10^6^ cells/mL) were cultured in 25-cm^2^ flasks and treated with 0 (control) 20, 40 (the IC_50_ value), or 80 µM asiaticoside for 48 h. Total RNA was extracted with the Ultraclean Tissue RNA Isolation Kit (Mo Bio, USA) from cells according to the manufacturer’s protocol using the global microarray analysis (Agilent Technologies, USA). Then, cDNA was synthesized from the isolated RNA using the first-strand cDNA synthesis by the RevertAidTM First Strand cDNA Synthesis Kit according to the manufacturer’s protocol. The expression levels of inflammation-related genes including interleukin-6 (IL-6) and tumor necrosis factor-α (TNF-α) were examined. The following primer sequences were used: for IL-6 the forward primer was, 5′-TTGCCTTCTTGGGACTG-3′, and the reverse primer was, 5′-CTGGCTTTGTCTTTCTTGTTA-3′, and for TNF-α the forward primer was, 5′-GTCGTAGCAAACCACCAAG-3′, and the reverse primer was, 5′-GTCGCCTCACAGAGCAAT-3′. The internal standard was used as the GAPDH forward primer, 5′-ACCACAGTCCATGCCATCAC-3′, and for the reverse, 5′-TCCACCACCCTGTTGCTGTA-3′. The 7500 Real-Time PCR System (Applied Biosystems, USA) was set up to measure these primers. Briefly, the system was performed using 45 cycles at 95 °C for 20 s with a specific annealing temperature for 4–5 s and 72 °C for 8 s. Amplification specificity was checked using a melting curve following the manufacturer’s instructions.

### 3.7. Detection of Apoptosis Using ^99m^Tc-Labeled Annexin V and MIBI

To assess tumor growth and early apoptosis after asiaticoside treatment, the MCF-7 cells (1 × 10^6^) were incubated with 40 µM asiaticoside for 48 h. The uptake of 60 MBq of ^99m^Tc-Labeled-annexin V or ^99m^Tc-Labeled-MIBI each for 30 min was measured with a dose calibrator (ATOMLAB 100) and according to a previously reported procedure [16]. The cells were washed 3 times with ice-cold phosphate-buffered saline and incubated with a nonradioactive medium. The efflux of radioactivity in the medium was measured. The cells then were trypsinized and centrifuged. Cells were solubilized with 1% sodium dodecyl sulfate and radioactivity uptake was recorded.

### 3.8. Experimental Animals and Ethics Statement

A protocol for using small animals was approved by the Kuwait Institutional Review Board of Animal Care ethics committee and used at the Faculty of Medicine–Animal Resources Centre (ARC) of Kuwait University (project number MN01/09). The ethics committee approval number was 132003 in the year 2018. The mice were injected with a dose of 150 mg/kg asiaticoside (0.5 mL) dose by an intraperitoneal injection [21,22,23].

### 3.9. The General Care of the Animals

Athymic nude mice-BALB/c congenic of 4 weeks of age (22 ± 5 g) were purchased from a commercial breeding company (Charles River, Hungary) and were provided with free access to standard mouse food, water, and housing under specific pathogen-free conditions. The environment of the animal room was carefully controlled, with a 12-h dark/light cycle, a temperature of 20 °C, and a humidity of 45%. 

### 3.10. Animal Experiments

**Figure 10:**
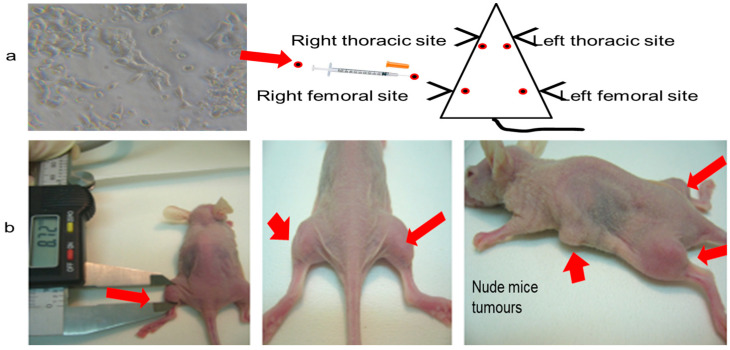
Photographs of (**a**) MCF-7 cells injected into four sites in a nude mouse and (**b**) a nude mouse and its palpable tumors (red arrows).

Mice were randomly divided into five groups (n = 10 per group, Table 3) and studied for 8 weeks. Group I (control) comprised mice that were treated with an intraperitoneal injection of 0.5 mL saline. Group II (tumor induction) comprised mice subcutaneously injected with 2 × 10^7^ MCF-7 cells/ml saline. Four sites were injected in the left and right thoracic and left and right thighs, as shown in Figure 10. In group III (pre-treated), mice were prepared as group II but were intraperitoneally injected with asiaticoside once per each week of the 8 weeks study. In group IV (post-treatment), mice were subcutaneously inoculated with 2 × 10^7^ MCF-7 cells as stated above and intraperitoneally injected with asiaticoside starting at week 6. Finally, group V (drug control) mice were only intraperitoneally treated with asiaticoside once per week from week 1 to week 8. Weight measurements were performed weekly for all mice in each group. This was conducted before and during the experiment.

### 3.11. Experimental Protocol

Mice were randomly divided into five groups (n = 10 per group, Table 3) and studied for 8 weeks. Group I (control) comprised mice that were treated with an intraperitoneal injection of 0.5 mL saline. Group II (tumor induction) comprised mice subcutaneously injected with 2 × 10^7^ MCF-7 cells/mL saline. Four sites were injected in the left and right thoracic and left and right thighs, as shown in Figure 1. In group III (pre-treated), mice were prepared as group II but were intraperitoneally injected with asiaticoside once per each week of the 8 weeks study. In group IV (post-treatment), mice were subcutaneously inoculated with 2 × 10^7^ MCF-7 cells as stated above and intraperitoneally injected with asiaticoside starting at week 6. Finally, group V (drug control) mice were only intraperitoneally treated with asiaticoside once per week from week 1 to week 8. Weight measurements were performed weekly for all mice in each group. This was conducted before and during the experiment.

When the tumors became palpable, measurements of tumor volume were performed (with calipers) once per week. The tumor volume calculation was executed using the formula V = (W2 × L)/2 for caliper measurements, where V is tumor volume, W is tumor width, and L is tumor length [24].

At the end of week 8, all animals were sacrificed by CO_2_ euthanasia (CO_2_ inhalation). A gradual fill of CO_2_ exposure with a displacement rate from 30–70% of the euthanasia chamber volume/min was performed using a flowmeter (1.3 L/min). This was followed by cervical dislocation to ensure the death of the animal. Then, tumors were excised. After tumor removal, the weights of the tumors were determined to be divided into 2 halves: one half was stored for histology in buffered (10%) neutral formalin, followed by haematoxylin and eosin (H and E) staining, and the other half was frozen for DNA and RNA isolation and further evaluations.

### 3.12. Real-Time (RT)-PCR Analysis of Gene Expression in Tumours

All analyses evaluated the same mice. For gene expression analyses of tumor tissues, an amount of 50 mg of minced tumor tissue was homogenized in 1 mL of one-step RNA reagent containing 0.25 mg glycogen for 1 min at 15–20 s intervals using a sonicator (Fisher Scientific, USA). Then, the total RNA was isolated from the tumor tissues using TRIzol and a one-step RNA isolation kit (Medox Biotech, India) according to the manufacturer’s instructions. The purity and concentration of the RNA isolated from the homogenates were measured and determined. The RNA was assessed using UV spectroscopy. The absorbance of a diluted RNA sample was measured at 260 and 280 nm.

### 3.13. Western Blot Analysis

An amount of 500 μL RIPA buffer for every 10 mg of tissue was added, homogenized thoroughly using a sonicator (Fisher Scientific, USA), and kept on ice for 30 min. Then, lysates were centrifuged in a microfuge at the 15,000 RPM speed for 20 min at 4 °C to pellet cell debris and were then transferred to the supernatant to a fresh microfuge tube without disturbing the pellet.

For each group, the protein concentration 10 µL of each sample was determined with a Bio-Rad expression and purification kit (Bio-Rad Laboratories, UK). Proteins were separated by gel electrophoresis SDS-PAGE gel and transferred to polyvinylidene difluoride (PVDF) membranes. The membranes were blocked with 3% bovine serum albumin (BSA) for 2 h at room temperature to avoid non-specific binding. Then, the membranes were incubated with the indicated primary antibody, the rabbit polyclonal antibody, Cat. No. PA5-17848, 1:1000 dilution, for 24 h at 4 °C. The membranes were washed with PBS to remove any unbound primary antibody and incubated with goat anti-rabbit IgG, secondary antibody (Cat. No. A27036, 0.25 µg/mL, 1:4000 dilution) for 30 min at 4 °C, followed by three washes with PBS. β-actin was used as a control. Protein bands were visualized using the ChemiDoc MP Imaging System and Image Lab software (version 5.1). 

### 3.14. Ribonucleic Acid (RNA) Purification and Microarrays

RNA samples with higher quality and yield were extracted by the modified TRIzol method from MCF-7 cells and tumors following the manufacturer’s protocol with a few modifications. The incubation steps at 55 °C and at 80 °C were reduced to decrease the RNA degradation. The RNA quality was confirmed by analysis with an Agilent 2100 Bioanalyzer.

Microarray analyses were conducted using the Agilent microarray platform. An amount of 350 ng of total RNA was reverse-transcribed into the first strand and second strand cDNA. Microarrays were scanned using an Agilent G2505B Scanner controlled by Agilent Scan Control 7.0 software. 

### 3.15. Statistical Analysis

Data are presented as means ± standard deviations (SDs). A paired t-test was used to compare the control and treated groups in both the cell and mouse experiments. A *p*-value equal to or less than 0.05 was considered to indicate a significant value. Statistical analysis was performed using SPSS version 18.0 (SPSS Inc., USA).

## 4. Discussion

This study aims to examine the anti-tumor effects of asiaticoside on breast cancer, to understand the mechanisms of action of asiaticoside and the pathways of inflammation in MCF-7 cells and in an MCF-7 xenograft nude mouse model. In this study, the half maximal inhibitory concentration (IC50) 40 µM of asiaticoside was used as it was reported previously [9]. In this study, the results showed that asiaticoside increased caspase-9 activity and decreased both TNF-α and IL-6 expression via the NF-κB pathway. Our results showed that asiaticoside administration significantly increased caspase-9 activity in MCF-7 cells and in nude mouse xenografts (in III and IV group) compared to controls. Caspase-9 is an initiator caspase and acts on the intrinsic or mitochondrial pathway, which is involved in chemotherapies, functional changes, or cancer development [25]. Many studies reported that caspase-9 activity initiates and triggers intrinsic apoptosis [26,27,28,29]. To rule out the inhibition of caspase-9, here caspase9 protein expression was measured in the xenograft tumors. This involved an in vivo study to explain more about the mechanism of asiaticoside. Caspase-9 protein expression was shown in all studied groups in Western blot assays. At the same time, asiaticoside treatment decreased the gene and protein expression of inflammatory cytokines in treated xenograft mice in our study. It was shown that asiaticoside has intense anti-inflammatory effects. This agrees with many studies whch used various molecular and biochemical methods that proved that asiaticoside can suppress the release of pro-inflammatory biomarkers, including IL-1β, IL-6, and TNF-α [9,30,31,32,33] and induce apoptosis [34] by arresting the cell cycle in S phase [9]. This was proved by the ELISA, Western blot and the DNA binding assay results. The mechanism of asiaticoside has rarely been reported.

Our study highlights the effect of asiaticoside on apoptosis in MCF-7 cells and nude mice. Asiaticoside promotes apoptotic signaling in MCF-7 cells and tumors, prevents tumor growth, and decreases inflammatory activity.

In this study, IL-6 and TNF-α were reduced and inhibited. Many studies reported that IL-6 is an essential component for performing many functions such as the proliferation, metastasis, and hormonal regulation functions of cancer cells. IL-6, for example, regulates estrogen production in breast cancer cells and tissues [35]. One study showed that IL-6 down-regulation expression leads to growth inhibition of MCF-7 [36].

Moreover, IL-6 is one of the major proinflammatory cytokines that play a key role in the growth, invasion, and metastasis of MCF-7 cells [37]. In the present study, IL-6 was reduced after asiaticoside treatment in breast cancer cells. This is in agreement with studies that reported decreased IL-6 levels due to therapy [38] and a relapse of body weight loss in complicated breast cancer patients [35].

TNF-α, similarly to IL-6, is a proinflammatory cytokine characterized by a broad spectrum of functions which also include cytotoxic and cytostatic effects against cancer cells and in MCF-7 cells [39]. TNF-α and IL-6 are reported to influence each other’s expressions. Some studies pointed out the direct relation between TNF-α and tumor growth [10,11,12,40,41,42]. Our stfudy supports the previous reports on the expression of these pro-inflammatory cytokines in MCF-7 cells [43] and showed that asiaticoside significantly reduced IL-6 and TNF-α. This indicates that asiaticoside can reduce inflammation or prevent the development of inflammatory diseases.

NF-kB exerts many actions in the process of inhibition of apoptosis, proliferation, and many responses such as stress, inflammation, and immunity [44]. We suspected a relationship between the working mechanism of asiaticoside and its inhibition of NF-kB. The subcellular fractionation was performed to measure protein levels in the nuclear and cytosolic fractions of MCF-7 cells and tumor xenografts per study group. The DNA binding assay was performed. In addition, NF-kB acts at the crossroads of many signaling pathways. Inappropriate or excessive activation of NF-kB can result in inflammatory diseases and tumors [45]. In this study, asiaticoside downregulated the NF-κB pathway.

Asiaticoside inhibited the degradation of IκB and IKK. A study showed that some drugs inhibited NF-κB activation [46]. This was associated with the DNA binding and IκB [47].

Our results agree with a study which reported that asiaticoside has suppressed the inflammatory responses in lipopolysaccharide-induced acute lung injury through the deactivation of the phosphorylation of NF-kB and the degradation of its inhibitor IκBα [48]. Furthermore, one study reported that asiaticoside suppresses cell proliferation by inhibiting the NF-κB signaling pathway in colorectal cancer [30].

The inhibition of NF-κB signaling may provide an effective method to inhibit cell proliferation, growth, and apoptosis inducement. All tumor-bearing nude mice developed tumors at the injection sites. Tumor growth was regressed and decreased after asiaticoside treatment. In addition, the body weight was monitored and recorded to investigate the study effect on the animals. The uptake of ^99m^Tc-labeled Annexin V and MIBI confirmed the detection of early apoptosis after asiaticoside treatment. Annexin V and MIBI have a high sensitivity and specificity (>90%) for apoptosis and breast cancer, respectively.

The ability of asiaticoside to regress established tumors and the effect of its chemopreventive treatment could be the result of the observed inhibition of the NF-κB pathway activation.

Many studies reported that other chemopreventive agents such as non-steroidal anti-inflammatory drugs (NSAIDs) and aspirin are only beneficial for colonic adenomas [49]. The use of pentagamavunon-1, a curcumin analog that exhibits anti-cancer properties toward several cancer cells; curcumin against various cancer cells; and epigallocatechin-3-gallate in certain conditions were reported [50]. Apigenin exerts anti-tumor effects mainly by inducing apoptosis/cell-cycle arrest [51] However, asiaticoside surpasses other chemopreventive agents not only by inhibiting the proliferation, epithelial-mesenchymal transition, and stem cell-like properties of different cancers: it also overcomes drug-resistant cancer [11], chemo-resistance, and toxicity and serious side effects caused by long-term use [29,52].

The most common cancer in the genitourinary system is breast cancer. When patients are diagnosed, 30% have distant metastases and 25% have localized disease. In total, 61.7% of patients with the locally advanced disease survive for 5 years. In the metastatic stage, BC has a 5-year survival rate as low as 9%. The vast majority of BC patients either relapse after an initial response to immunotherapies or are resistant to these treatments. Recent tyrosine kinase inhibitors, sorafenib and sunitinib, have demonstrated modest activity against BC, but patients suffer hepatotoxicity and other side effects. It is vital to develop new therapeutic strategies for BC because, since asiaticoside has less toxicity, they are promising for cancer treatment.

In our study we have many limitations. For example, we have used one cell line that might have influenced the results, particularly considering that MCF-7 cells do not typically show metastatic activity in mice. The results may be different if we can use a more metastatic cell line. In future studies, we will use a panel of cell lines that might provide further confirmation of our results. In addition, we will study if asiaticoside can treat other types of cancers.

## 5. Conclusions

In conclusion, this study confirmed that asiaticoside significantly decreased IL-6 and TNF-α in MCF-7 cells and xenografts via the caspase-9—NF-κB pathway. This supports asiaticoside inhibitory abilities in breast cancer survival and progression. Further investigation regarding the clinical role of asiaticoside is required.

## Figures and Tables

**Table 1:** Xenograft tumor growth: tumor volume in mm^3^ in all groups and 10 mice per group. Four sites were injected in each mouse. Left sites relating to tumors are summarized in Table 1. In groups II, III, and IV, all of the injected tumours grew, which allowed us to assess 120 tumours in total. Data is expressed as mean ± SD.

Time (Week)					
Tumour Site	4	5	6	7	8
II Thoracic	3.66 ± 0.5	5.34 ± 0.3	36.12 ± 0.08	70.04 ± 0.4	130.32 ± 0.7
II Femoral	3.78 ± 0.6	29.42 ± 0.4	159.58 ± 0.6	278.98 ± 0.9	787.9 ± 0.8
III Thoracic	0 ± 0.0	2.08 ± 0.03	9.08 ± 0.07	30.04 ± 0.3	94.98 ± 0.6
III Femoral	0.84 ± 0.03	8.98 ± 0.2	15.97 ± 0.05	150.98 ± 0.7	273.09 ± 0.9
IV Thoracic	0 ± 0.0	18.95 ± 0.3	45.89 ± 0.7	65.98 ± 0.8	109.04 ± 0.8
IV Femoral	3.89 ± 0.4	42.97 ± 0.5	189.96 ± 0.8	396.94 ± 0.9	670.02 ± 0.9

**Table 2:** TNF-α and IL-6 levels of tumor tissue in the control and groups II, III, IV, and V. Experiments were repeated three times and data are presented as mean ± SD. * *p* < 0.05; ** *p* < 0.01; and *** *p* < 0.001 unpaired Student *t*-test.

Group	I	II	III	IV	V
TNF-α (pg/mg protein)	124.34 ± 5.21	297.31 ± 7.81 ***	167.33 ± 2.68 **	176.95 ± 4.38 *	112.36 ± 5.31
IL-6 (pg/mg protein)	152 ± 12.67	375 ± 19.35 ***	210.52 ± 4.89 **	255.34 ± 3.67 *	143.57 ± 4.2

**Table 3:** The mice were injected with a dose of 150 mg/kg asiaticoside (0.5 mL) intraperitoneally.

Time (Week)								
Group	1	2	3	4	5	6	7	8
I	Saline	Saline	Saline	Saline	Saline	Saline	Saline	Saline
II	Saline	Saline	MCF-7 cell injection	Saline	Saline	Saline	Saline	Saline
III	Asiaticoside	Asiaticoside	MCF-7 cell injection	Asiaticoside	Asiaticoside	Asiaticoside	Asiaticoside	Asiaticoside
IV	Saline	Saline	MCF-7 cell injection	Saline	Saline	Asiaticoside	Asiaticoside	Asiaticoside
V	Asiaticoside	Asiaticoside	Asiaticoside	Asiaticoside	Asiaticoside	Asiaticoside	Asiaticoside	Asiaticoside

## Data Availability

The data presented in this study is contained within the article.
